# Association between Urinary Triclosan and Serum Testosterone Levels in U.S. Adult Males from NHANES, 2011–2012

**DOI:** 10.3390/ijerph17207412

**Published:** 2020-10-12

**Authors:** Judy Yan, Michael A. Joseph, Simone A. Reynolds, Laura A. Geer

**Affiliations:** 1 Department of Environmental and Occupational Health Sciences, SUNY Downstate Health Sciences University, Brooklyn, NY 11203, USA; jyan3268@gmail.com; 2 Department of Epidemiology and Biostatistics, SUNY Downstate Health Sciences University, Brooklyn, NY 11203, USA; michael.joseph@downstate.edu (M.A.J.); simone.reynolds@downstate.edu (S.A.R.)

**Keywords:** antimicrobial, triclosan, human exposure, endocrine-disruptor, testosterone, NHANES, antibacterial

## Abstract

Triclosan was introduced into the market in the 1970s and has since been used as an antimicrobial agent in a diverse array of consumer and personal care products. Although it has been widely used over a number of years, there is growing concern and debate over its safety and efficacy and its potential as an endocrine disruptor. Although prior animal toxicology studies have shown an association between triclosan and decreased testosterone levels, human studies have been limited, particularly for adult men. Using the National Health and Nutrition Examination Survey data (NHANES, 2011–2012), we examined the association of urinary triclosan on testosterone levels in adult men 18–65 years of age. Multivariable linear regression analysis failed to show an association between triclosan and serum testosterone (β = 0.0003, *p* = 0.98, 95% CI = −0.024, 0.025). The results suggest there is no association or that triclosan concentrations are too low to cause a significant impact on testosterone levels. Additionally, longitudinal studies would provide a more comprehensive understanding of the direction of change and magnitude of causal relationships over time.

## 1. Introduction

Triclosan is a broad-spectrum antimicrobial agent found in many personal care and household products [1]. The ubiquitous use and potential bioaccumulation of triclosan have led to detectable levels in aquatic ecosystems as well as in human tissues and fluids [2]. As a result, concerns and debate over its potential as an endocrine disruptor and impact on human health continue to grow. Various studies have been conducted on the impact of environmental chemical exposure on the male reproductive system. Several of these studies have shown significant associations with endocrine disruptors such as polycyclic aromatic hydrocarbons and phthalates, and decreases in testosterone levels and semen quality [3,4,5,6]. In humans, long-term exposure to endocrine-disrupting chemicals has been shown to have harmful effects on reproductive health by interfering with the development and function of sex hormones [7,8]. Studies on animals have shown similar results. In rat models, exposure to triclosan has led to the disruption of the biosynthesis of testosterone levels [9,10,11]. In addition to being an important male sex hormone, testosterone plays an important roles in health and disease pathology. In humans, low testosterone levels are associated with an increased risk of mortality from all causes [12]. Even slight disruptions in circulating levels of testosterone can adversely impact human health [13] and can be a risk factor for common medical conditions such as metabolic syndrome, obesity, diabetes, hypertension, and atherosclerosis [14,15]. Low testosterone levels have also been associated with depression [16]. Analyzing data from the 2011–2012 National Health and Nutrition Examination Survey (NHANES), we recently reported no association between triclosan levels and white blood cell counts [17]. To broaden our understanding of triclosan, with this current study, we aimed to assess the potential health risks of triclosan and its hormone-disrupting potential, particularly on serum testosterone levels in human adult males. Although several studies [3,7,8] on the effects of triclosan and serum testosterone levels have shown adverse impacts on reproductive health, one study on children and adolescents aged 6–19 years examined the association of several endocrine-disrupting compounds and their anti-androgenic effects but found little association between triclosan and testosterone levels [18]. Based on the evidence of previous research findings, we hypothesized that triclosan is associated with decreased serum testosterone levels in adult men.

## 2. Materials and Methods

### 2.1. Sample Selection

To evaluate the association between triclosan and total testosterone levels in U.S. adult men aged 18–65 years, laboratory and survey data from NHANES 2011–2012 were examined. The NHANES is an annual cross-sectional survey using interviews, examinations, and laboratory data to assess the health and nutritional status of the general, non-institutionalized U.S. population. Its data are used to estimate the prevalence of select diseases and health risk factors. The presence of triclosan in urine is indicative of absorption and systemic exposure [19]. Because triclosan is mainly excreted in urine [20], we examined urinary triclosan concentrations. For concentrations below the level of detection, NHANES imputes triclosan values equal to the detection limit divided by the square root of 2. Total testosterone was measured in serum. The validity, reliability, and quality control of laboratory data are detailed in the NHANES Laboratory Procedure Manual [21]. For this study, the 18–65-year age group was chosen because testosterone levels vary by age. Children younger than 10 years have low testosterone levels, which increase between 10 and 15 years with the onset of puberty [22]. Total testosterone peaks at an average age of 19 years [23]. As men age, testosterone levels decline, particularly in older men above 65 years [24]. Therefore, to limit the confounding effects of age, we restricted the study to men between 18 and 65 years.

**Figure 1:**
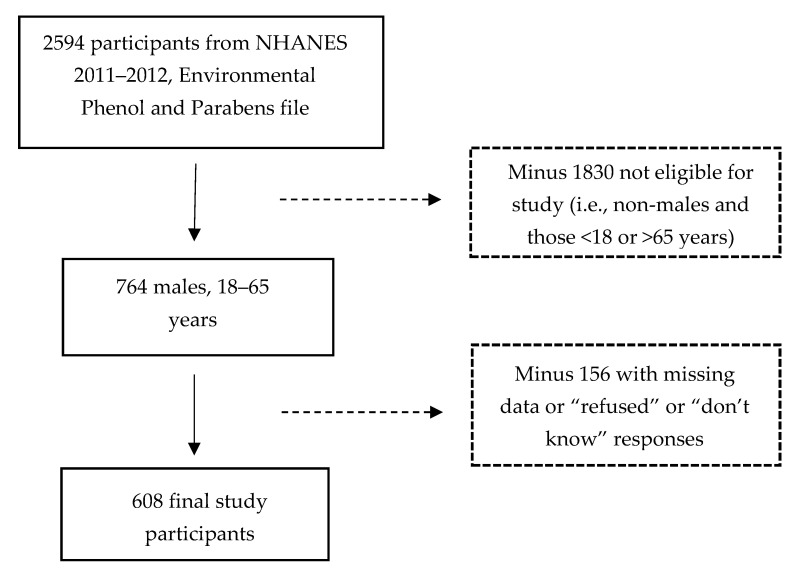
Selection of study sample. NHANES, National Health and Nutrition Examination Survey.

To improve the reliability of the health and nutrition estimates, the NHANES study oversampled the subpopulations of Hispanic and non-Hispanic Black participants. Oversampling of non-Hispanic Asian participants occurred during the 2011–2012 wave. Among the 9756 (weighted *N* = 306,590,681) individuals who participated in the 2011–2012 survey, data on the urinary biomarkers of triclosan exposure were collected on a subsample of 2594 participants (weighted *N* = 282,460,101). All analyses were conducted on this subsample. We first restricted our study sample to men aged 18–65 years (*n* = 764). Participants with missing data (including “refused” or “don’t know” responses) for variables used in the multivariable model were excluded (*n* = 156). The final sample for this study consisted of 608 survey participants (weighted *N* = 80,795,632). Of the 156 who were excluded from the study because of missing observations, 9 had non-positive weights. The 156 respondents did not differ from the remaining 608 in triclosan levels, age, and creatinine levels (all *p* > 0.05). The percentage distribution between participants and non-participants was relatively similar for smoking (26.8% vs. 24.5% respectively, *p* = 0.59). The participant group had a larger proportion of high-income Whites than the non-participant group (income (low: 19.0% vs. 21.6%, medium: 37.5% vs. 23.5%, high: 43.5% vs. 11.3%), *p* < 0.05; race categories (White: 67.0% vs. 46.3%; Mexican-American/Hispanic/Other: 18.9% vs. 22.7%, Black: 9.6% vs. 21.4%, Asian: 4.5% vs. 9.6%,), *p* < 0.05). Compared with the 156 non-participants who were excluded, the means among the 608 with complete data on predictors were statistically significantly higher for serum testosterone (415.1.5 ng/dL vs. 178.9 ng/dL, *p* < 0.0001) and body mass index (BMI) (28.7 kg/m^2^ vs. 26.4 kg/m^2^, *p* < 0.02). Figure 1 shows the process of selecting the study sample for analysis.

### 2.2. Confounding Measures

The selection of covariates to control for as confounders was determined through a review of the existing literature. Triclosan exposure has been shown to vary by race, BMI, family income [25,26,27], and creatinine levels [28,29]. Triclosan concentrations have also been associated with lifestyle factors such as cigarette smoking [25,30,31]. It is suggested that certain lifestyle factors and choice of personal care products affect varying levels of triclosan exposure. For this study, race was categorized as: Black, White, Asian, and Mexican-American/Hispanic/Other, with the last category combined due to smaller numbers. Because the categorization of BMI can bias results and lead to loss of information [32,33,34], BMI was investigated as a continuous variable. Annual household income was stratified as “low income” (<25th percentie), “middle income” (25th–75th percentile), and “high income” (>75th percentile). To reduce the potential for recall and/or self-reporting biases, smoking status was based on urinary cotinine as a biochemical marker of tobacco smoke exposure. Survey respondents were biochemically classified as smokers or non-smokers based on the thresholds outlined by the Centers for Disease Control and Prevention (CDC) (2013) guidelines [35] where serum cotinine levels were either lower than 10 ng/mL (non-smoker) or 10 ng/mL or greater (smoker). Although the use of binary thresholds for serum cotinine levels cannot definitively discriminate between smokers and non-smokers, one study [36] reported the optimal cutoff for serum cotinine levels of 10 ng/mL is generally accepted. Because the predominant route of triclosan excretion is via urine, differences in the dilution of urine can affect the concentration of triclosan. Additionally, urine flow rate can strongly vary over time, as well as within and among individuals [37]. Creatinine excretion in urine, however, occurs at a less variable rate than the rate of urinary flow. Therefore, creatinine levels (mg/dL) were adjusted as an independent variable to correct for differences in urinary concentrations of triclosan. Table 1 details the description of the variables used in this study.

## 3. Statistical Analysis

The normal distribution of continuous variables was evaluated by visually interpreting the normality plot and by assessing the skewness and kurtosis values. Variables with skewness or kurtosis values that deviated from the acceptable range of –1 to 1 for a normal distribution were log-transformed to approximate normality. All continuous variables required log-transformation, which was applied prior to regression analyses. The estimated association between serum testosterone and triclosan levels was performed using multiple linear regression. Appropriate confounders for the regression model were identified based on the change-in-estimate criterion, in which controlling for a covariate alters the regression coefficient for the unadjusted univariate model by more than 10% [38]. Potential multicollinearity among the predictor variables was assessed by examining the variance inflation factors (VIF) and tolerance (the inverse of VIF) diagnostic statistics. Although there is no formal VIF value for detecting multicollinearity, values of VIF 10 or above and tolerance below 0.1 are often indicative of multicollinearity [39]. The potential for bias arising from missing data between the participants of the study with complete observations and those excluded from the study because of missing values was assessed using *t*-tests for continuous variables; categorical variables between these two groups are presented as weighted percentages and compared using the Rao–Scott adjusted chi-square test [40,41]. As recommended by the National Center for Health Statistics (NCHS) [42], all analyses for this study used appropriate weights to account for the complex sampling design in the NHANES data to ensure that the calculated estimates reflected a nationally representative sample of the U.S. population. Analyses were conducted using SAS Enterprise Guide 6.1 (Cary, NC, USA) with the statistical significance level set at α = 0.05.

## 4. Results

Table 2 shows the weighted mean serum testosterone and triclosan levels for the final 608 study participants. The weighted mean age of the participants in the study was approximately 41 years, with a BMI of 28.7 kg/m^2^. Mean triclosan levels were found to be highest among those of Mexican-American/Hispanic/Other race (mean = 162.0 ng/dL, standard deviation (SE) = 36.8 ng/dL, *p* < 0.0004), those in the high-income category (mean = 135.8 ng/dL, SE = 47.1 ng/dL, *p* < 0.0104), and those classified as non-smokers (mean = 137.0 ng/dL, SE = 36.1 ng/dL, *p* < 0.0015). Weighted mean serum total testosterone levels did not differ across race, income category, or smoking status. The results showed that the overall multivariable regression model significantly predicted serum testosterone levels and indicated that the predictors used in the model explained ~16% of the variance in serum testosterone levels (*R*^2^ = 0.163, *F*(6, 17) = 17.1, *p* < 0.0001). Body mass index was the only predictor found to be significant (β = –0.868, *t* = –5.99, *p* < 0.0001); see Table 3.

Statistically significant confounders included BMI, smoking, and income category. Creatinine was adjusted for as a covariate in the regression model to account for urinary dilution [28]. Race and age were not significant confounders in the bivariate model. Tolerance and VIF values did not provide evidence of multicollinearity among the predictor variables (VIF 1.04–1.09; tolerance all > 0.1).

Our study did not find evidence of an association between triclosan and male serum testosterone levels (β = 0.0003, *p* = 0.98, 95% CI = −0.024, 0.025).

## 5. Discussion

Using a nationally representative sample of the U.S. population, we did not find a statistically significant association between total serum testosterone and urinary triclosan levels in adult men. Contrary to previous research on animals, the results from our study do not corroborate animal toxicology findings that suggest a downregulation in testicular testosterone levels in rats treated with dose-dependent triclosan levels [9,10,11]. Additionally, a cross-sectional study of children and adolescents [18] found an inverse association between triclosan and serum testosterone levels, although statistical significance was not consistent across children or adolescents of either sex. Our results may indicate that correlations do not occur or that urinary triclosan concentrations may be too low to have an acute impact on testosterone levels. Nonetheless, a larger and statistically significant relationship may emerge after longer use of or higher-dose exposure to products containing triclosan. Although this current study did not provide evidence of an association, prior studies are consistent with the current biological understanding of triclosan’s adverse effect on serum testosterone levels, suggesting the biological plausibility of a role for triclosan as a potential androgen-disrupting chemical.

The present study has several limitations. The analysis has potential for selection bias, as the decision to participate in the NHANES survey may reflect underlying differences in the respondents. Possible bias from exclusion of participants from analysis due to survey non-response and missing values for some variables also exists. Additionally, the cross-sectional nature of this study restricts interpretations of causality between triclosan and testosterone. Reverse causation cannot be ruled out as a potential explanation of the findings, as the amount of triclosan found in the urine may be the effect, not the cause, of variation in testosterone levels. Finally, data for factors such as stress levels, time of day when the serum testosterone levels were taken, or the temporal proximity of sexual activity were not available but may potentially have had some level of impact on the association between triclosan and testosterone [43,44,45].

Despite the limitations, one of the strengths of this study is the rigorous quality assurance and controlled measures in the NHANES data. Measurement bias was also reduced, as urinary concentrations of triclosan and serum testosterone levels were based on validated laboratory methods. Future efforts could include longitudinal study designs to further explore the associations and determine whether triclosan exposure can alter testosterone levels in adult human men. Potential confounders to consider could also include dietary habits, various other hormone levels, medications used, alcohol consumption, and history of exposure to certain chemicals or heavy metals such as mercury or lead.

## 6. Conclusions

After decades of triclosan being in the consumer market, the U.S. Food and Drug Administration (FDA) issued a final rule in September 2016 banning 19 antimicrobial agents, including triclosan, in many commonly used consumer antiseptic wash products [46]. The final rule, however, is limited. It only applies to hand and body soaps, whereas triclosan-containing products used in institutional settings such as hospitals and food preparation areas are exempt. Scientists and researchers have raised concerns about the widespread use of antimicrobial agents and have called for stricter regulations worldwide. The Florence Statement on Triclosan and Triclocarban (2017) [47], a consensus statement of over 200 signatories from 29 countries with expertise on the health and environmental impacts and efficacy of antimicrobials, documented the hazards and lack of health benefits associated with the use of triclosan, triclocarban, and other antimicrobials. In light of the research on the adverse effects of triclosan, some manufacturers such as Procter & Gamble, Johnson & Johnson, and Avon have voluntarily removed triclosan from their products. However, triclosan can currently still be found in products unregulated by the FDA such as plastics and textile products including toys, clothing, kitchenware, and furniture [48,49]. Considering the potential harm triclosan is known to cause to human and animal health, continual surveillance in longitudinal studies is a prudent approach to capture any possible long-term impacts of triclosan exposure on hormone levels, including testosterone, in the human body.

## Figures and Tables

**Table 1:** Description of study variables.

Category of Variable	Variable Name	Type of Variable	Description
Response variable	Total serum testosterone (ng/dL)	Continuous	Lab values
Main predictor variable	Urinary triclosan (ng/mL)	Continuous	Lab values
Covariates	Race	Categorical	Black, White (reference), Asian, Mexican-American/Hispanic/Other
	Body mass index (kg/m^2^)	Continuous	Lab values
	Annual income	Categorical	Low income (≤ 25th percentile),
			Middle income (25th–75th percentile)
			High income (≥ 75th percentile)
	Cotinine levels (ng/mL) (as a proxy for smoking status)	Binary	Non-smoker (lower than 10 ng/mL)
			Smoker (10 ng/mL or greater)
	Creatinine levels (mg/dL)	Continuous	Lab values

**Table 2:** Weighted mean levels [standard deviation (SE)] of urinary triclosan and total Serum testosterone by potential confounders.

				Weighted Mean (SE)					
**Independent Variables**	**Categories**	**Unweighted *N* (%)**	**Weighted *N* (%)**	**Mean**	***p***	**Urinary Triclosan, ng/mL**	***p***	**Total Serum Testosterone, ng/dL**	***p***
**Age, years**	N/A	608		40.7 (0.96)	<0.0001	112.0 (24.7)		415.1 (12.5)	
**BMI, kg/m^2^**			80,795,632	28.7 (0.36)	<0.0001		0.0003		<0.0001
**Creatinine, mg/dL**				137.6 (5.55)	<0.0001				
**Race**									
	Mexican-American/Hispanic/Other	149 (24.5%)	15,215,210 (18.8%)			162.0 (36.8)	0.0004	413.6 (11.2)	<0.0001
	Black	154 (25.3%)	7,781,869 (9.6%)			49.4 (11.3)	0.0004	417.4 (17.8)	<0.0001
	Asian	93 (15.3%)	3,659,140 (4.5%)			79.5 (35.8)	0.0404	406.1 (16.4)	<0.0001
	White	212 (34.9%)	54,139,413 (67.0%)			109.1 (35.9)	0.0074	415.7 (17.2)	<0.0001
**Income**									
	Low	176 (29.0%)	15,354,875 (19.0%)			53.8 (18.2)	0.0089	421.0 (12.7)	<0.0001
	Middle	232 (38.2%)	30,292,907 (37.5%)			113.7 (29.2)	0.0012	433.6 (23.2)	<0.0001
	High	200 (32.9%)	35,147,851 (43.5%)			135.8 (47.1)	0.0104	396.5 (18.0)	<0.0001
**Smoking status**									
	Smoker	170 (25.0%)	21,638,149 (26.8%)			43.5 (10.5)	0.0007	432.9 (11.6)	<0.0001
	Non-smoker	438 (72.0%)	59,157,484 (73.2%)			137.0 (36.1)	0.0015	408.5 (16.2)	<0.0001

**Table 3:** Regression analysis for urinary triclosan and total serum testosterone levels, adjusted for significant covariates.

Independent Variables	Total Serum Testosterone Level		
	β-coefficient (SE)	95% CI	*p*
**Log (urinary triclosan)**	< 0.001 (0.012)	−0.024, 0.025	0.982
**Log (BMI)**	−0.868 (0.145)	−1.174, −0.563	<0.0001
**Log (creatinine)**	−0.025 (0.044)	−0.117, −0.067	0.579
			
**Smoking**			
**Smoker**	Reference	Reference	Reference
**Non-smoker**	−0.032 (0.044)	−0.124, 0.061	0.483
**Income categories**			
**Low**	−0.026 (0.049)	−0.129, 0.077	0.602
**Middle**	0.062 (0.053)	−0.051, 0.174	0.263
**High**	Reference	Reference	Reference
